# Executive functions and insight in OCD: a comparative study

**DOI:** 10.1186/s12888-021-03227-w

**Published:** 2021-04-29

**Authors:** Lucas Manarte, António R. Andrade, Linete do Rosário, Daniel Sampaio, Maria Luísa Figueira, Pedro Morgado, Barbara J. Sahakian

**Affiliations:** 1 Faculty of Medicine, University of Lisbon, Av. Prof. Egas Moniz, 1649-028 Lisbon, Portugal; 2 IDMEC. Instituto Superior Técnico, University of Lisbon, Av. Rovisco Pais 1, 1049-001 Lisbon, Portugal; 3 Life and Health Sciences Research Institute, School of Medicine, University of Minho, R. da Universidade, 4710-057 Braga, Portugal; 4 Department of Psychiatry and Behavioural and Clinical Neuroscience Institute, University of Cambridge, Herchel Smith Building for Brain & Mind Sciences, Forvie Site, Robinson Way, Cambridge, CB2 0SZ UK

**Keywords:** Obsessive-compulsive disorder, Insight, Wisconsin card sorting Test, Verbal memory, Trail-making test, Executive functions

## Abstract

**Background:**

Around 25 to 30% of patients with obsessive-compulsive disorder (OCD) do not respond to treatment. These patients have the longest duration of disease and the worst prognosis. Following years of research on this topic, insight has emerged as a potential explanation for this therapeutic resistance. Therefore, it has become important to characterize OCD patients with poor insight. Few studies have focused on the neuropsychological and cognitive characteristics of these patients.

**Methods:**

To help fill this gap, we divided 57 patients into two groups, one with good insight and the other with poor insight, assessed their neuropsychological functions—through a Rey’s figure test, a California verbal learning test, a Toulouse–Piéron test and a Wisconsin Card Sorting Test (WCST)—and compared the results with those of a paired control group.

**Results:**

The statistical analysis, with a significance level of 95%, revealed differences in the executive function tests, and particularly in the WCST (*p* ≤ 0.001) and trail-making-test (TMT A/B) (*p* = 0.002).

**Conclusions:**

These differences suggest that the neuropsychological profile of poor-insight patients is different from their good-insight counterparts, emphasize the role played by the executive functions in insight and highlights the need for more accurate neurocognitive research and treatment.

**Supplementary Information:**

The online version contains supplementary material available at 10.1186/s12888-021-03227-w.

## Background

Obsessive-compulsive disorder (OCD) affects 2% of adults worldwide and 10% of the adult clinical population [1]. Cognitive-behavioural psychotherapy is ineffective in 20% of cases and selective serotonin reuptake inhibitors (SSRIs) in 40% [2], although other studies suggest higher percentages for both treatments, around 40–60% [3]. Three factors can account for these low success rates: comorbidity, intensity of the symptoms and insight [4], with a prevalence of the latter [5–10]. Around 15–36% of OCD patients have poor insight [5, 11–16], which makes insight one of the most promising topics for future research in OCD [3, 17, 18].

Poor insight has been linked to many different factors: lower education levels [9], earlier age of disease onset, greater duration of disease [11, 15], chronic evolution and OCD family history [13] were the most cited. It has also been associated with more severe symptoms [5, 11, 12, 14] and a greater psychiatric comorbidity [9, 11–15].

The notion of insight has been the object of controversy and is viewed since 1990 as a multidimensional construct [19, 20]. On a psychopathological level, the connection between a lower insight in OCD and obsessive-compulsive symptoms has yet to be clarified [21]. Recent meta-analyses suggest that individuals with OCD have poorer verbal memory/fluency and visuospatial abilities/memory than healthy individuals and fare worse across several executive domains, such as sustained attention, processing speed and working memory [22]. However, OCD is a heterogeneous disorder, and these analyses do not specify which subgroup of OCD patients best fits this neuropsychological profile.

Executive functions are subserved by a neural network which includes regions of the frontal lobes [23, 24]. Executive functioning leads us to a set of functions or cognitive skills that, as defined in the dictionary of the International Neuropsychological Society, are “necessary to perform complex behaviors aimed at a certain objective” and determine our “adaptive capacity to different demands and environmental changes” [25]. These functions thus include a variety of adaptive skills and processes that allow the individual to “analyze what they want, how they might get it (ie form a plan, based often on recollections of past experience), and then carry that plan out”. Executive functions play an important and determining role in the individual’s cognitive, emotional and social regulation and, therefore, in the adoption of an effective, creative and socially acceptable conduct [26]. Given its nature and its role, it is not surprising that a commitment at this level can have a devastating impact on the person’s life, both in terms of the effectiveness of its daily functioning and in terms of the relationships it establishes with others. For this reason, it is important to have neuropsychological instruments to assess these functions. Executive functions include a wide range of cognitive processes, such as strategic working memory, cognitive flexibility, cognitive control of behaviour, planning and problem solving. Attention, the ability to anticipate, the establishment of objectives, the sequencing of activities, self-regulation and monitoring of behaviors, initiative, abstraction and spontaneity.

Since the prefrontal cortex has been identified by the specialized literature as the main neuroanatomical substrate for executive functions [26], much scientific knowledge has been produced in relation to the topic. Nevertheless, there are debates about issues related to the true nature of this construct, as well as the skills that integrate them. In this sense, several models of brain organization have been developed in order to explain the complex nature of these functions [25].

The present study aims to clarify whether poor-insight OCD patients have a different neuropsychological profile than their good-insight counterparts. To do so, and regardless of other clinical variables—such as age, sex, education, OCD symptoms (Yale–Brown Obsessive-Compulsive Scale, Y-BOCS), medication and insight (Brown Assessment of Beliefs Scale, BABS)—different aspects of neurocognition were analysed, namely: visual and verbal memory (Rey’s figure test, California verbal learning test, subjective memory questionnaire), visual attention and task switching (trail-making test), attention, working memory, visual processing, abstract reasoning, and the ability to change problem-solving strategies (Wisconsin Card Sorting Test), attention and perception (Toulouse–Piéron test).

The neuropsychological performance of OCD patients has drawn increasing attention [27, 28]. Since OCD patients were historically viewed as having average or above average intelligence [29], their performance deficits were not fully understood. However, the existing research has shown these patients have an average intelligence quotient (IQ), especially if one considers their verbal IQ but not their performance IQ.

It is widely accepted that OCD patients have a poorer performance in several neuropsychological dimensions compared to healthy controls, but the corresponding findings are heterogeneous, a sign that these patients are clinically diverse. Of the most significant neuropsychological findings observed to be compromised among OCD patients, we would like to highlight: attention, executive function, memory, visuospatial ablilities, processing speed and working memory and subdomains, as sustained attention, planning, response inhibition, set shifting/cognitive flexibility, verbal memory, non-verbal memory and spatial working memory [22]. There is a long debate concerning the reasons for these differences, and the type and severity of symptoms (assessed by the total Y-BOCS score) have been mentioned as possible explanations. In the last decades, however, despite the publication of different meta-analyses [30], no definite conclusion has been reached.

Poor insight may result from cognitive deficits and neuropsychological factors [31]: OCD patients with poor insight exhibited poorer speech learning and memory [32], fared worse in the trail-making test (TMT) and the Auditory-Verbal Learning Test (AVLT) than the group with better insight [33] and exhibited more severe neuropsychological deficits in executive functions [33], conflict resolution/response inhibition and verbal memory [28]. Nevertheless, very few studies have directly examined the specific neuropsychological alterations of OCD patients with poor insight [34]. Although the worse performance of patients with poor insight has yet to be conclusively explained, three reasons were advanced for their worse performance regarding specific cognitive deficits: 1) difficulties in the inhibition of the response-resolution conflict may prevent them from solving, in an adaptive manner, conflicts between their belief system and the corrective information they receive from the outside; 2) memory impairments may prevent from adequately updating their belief system; 3) fluency impairments may render the access to pre-existing memories more difficult [35].

Understanding the differences between insight groups in OCD can be very useful in the future. If we find, for instance, that neuropsychological profiles display significant contrasts depending on insight, which can be quickly evaluated in a routine psychiatric consultation, this discovery will allow us to determine which OCD patients merit a more detailed neuropsychological evaluation and to direct cognititive-behavioural therapy and psycho-education specifically towards the cognitive deficits mentioned in the previous paragraph.

In this study, we hypothesized that OCD patients with poor insight would have greater impairments in tests of executive functions, such as the WCST and TMT, compared with both OCD patients with good insight and healthy control subjects. As for memory tests, which require the use of a very different neural network, involving the hippocampus, we hypothesized that OCD patients with both good and poor insight would be equally impaired and show greater impairment as compared with the healthy control group.

## Methods

### Participants

Our sample consisted of 57 OCD patients, of which 34 men (59%) and 23 women (41%). We interviewed patients from the OCD Clinic/Braga Hospital and from the out-patient unit of Santa Maria Hospital diagnosed with OCD, according to DSM-IV TR. Eleven patients dropped out of the first interview, which led to a total of 110 (53 controls + 57 patients) fully evaluated participants. All patients had ages between 18 and 65 and gave their informed consent. Those with schizophrenia or other psychotic disorders, bipolar disorders, mental retardation, substance dependence, degenerative conditions or epilepsy were excluded from the study.

### Controls

The control group comprised the 53 blood donors from the Portuguese Blood Institute who best matched the clinical sample. From the total control participants, the outliers were removed and the remaining participants were included. Note that there were no significant differences between the OCD group and the control group (see Table 3), except for the variable “education years”, which, for this reason, was later controlled using ANCOVA. All controls were free from any psychiatric or other medical diseases/conditions.

### Measurements

All participants gave their informed consent and were asked to complete a two-phase interview. First, they were interviewed by a psychiatrist (the coordinator of this work). Afterwards, a neuropsychological evaluation was conducted by two trained neuropsychologists, who prepared together the agreed protocol and performed blind interviews (not knowing previously to which study group the interviewee belonged).

We conducted a semi-structured clinical interview including demographic data (age, birth date), data on medication (to determine whether participats regularly consumed psychotropic drugs, i.e. drugs belonging to the following pharmacologic groups: antidepressants, benzodiazepines, antipsychotics, mood stabilizers or amphetamines), years of education, Yale–Brown Obsessive-Compulsive Scale scores [36, 37] and the Mini International Neuropsychiatric Interview (MINI) [38]. MINI is a short-structured interview that assesses the criteria of the Diagnostic and Statistical Manual of Mental Disorders (4th ed.). MINI is considered a valid and time-efficient alternative to SCID-P (structured clinical interview for DSM-IV) and CIDI (composite international diagnostic interview), with kappa coefficients between 0.76 and 0.93 [38].

Insight was assessed using BABS. This scale is widely used because it allows for the comparison of different studies. Since it can also be used in a categorical manner [39], we established a cut-off point to distinguish poor insight (BABS score ≥ 12) from good insight (BABS score < 12). This allowed us to divide patients into four different categories, according to their total score: excellent (0–3), good (4–7), fair (8–12) and poor (13–17, or a total score of 18 plus a score of 0–3 on the conviction item). Furthermore, BABS considers a given belief to be delusional when the total score is 18 plus a score of 4 on the conviction item. In our study, poor insight refers to a total score > 12, while good insight corresponds to the other three categories put together (excellent, good, fair). This option agrees with the existing research on insight measured with BABS [40–44] and with our own experience using this scale to evaluate insight in OCD patients.

The English version of BABS was translated into Portuguese by a group of professional translators. The translation followed the standardized back-translation procedure to guarantee the semantic equivalence to the original [39]. This entailed two translations: one from the original version into Portuguese and another back into English. The second one was compared with the original to check for discrepancies and to reach a final consensus, which was then considered for the final Portuguese text. Each step of the process was carried out by a different translator. A confirmatory factor analysis was conducted to validate the one-factor structure of BABS using AMOS software (see Appendix I).

Neuropsychological evaluation: Besides the clinical evaluation, all participants were assessed with a battery of neuropsychological tests: California Verbal Learning Test (CVLT) [45, 46], trail-making-test (TMT) [47], Rey’s complex figure test [48–50], Toulouse–Piéron test (TBTP) [51], Wisconsin Card Sorting Test (WCST) [52]. All subjects were fluent in Portuguese and tested individually in a quiet testing room. The tests were mostly administered in the same sequence. The testing session for clinical subjects took approximately 120 min. In order to better detail the metris used, please see Table 1 with each test name as respective variables.

**Table 1:** Description, for each test, of the executive functions involved and the respective metrics [26]

Test name/measure definition	Description
Toulouse-Pierron test (TBTP)	It allows to evaluate the selective and sustained attention, as well as the processing speed, providing two main indices: work yield (RT) and dispersion index (ID).
TBTP-ID (dispersion index)	Allows us to understand if the result obtained on the work efficiency was influenced by a response pattern of impulsivity or innatention. This index is the percentage of errors and omissions divided by the number of hits obtained by the subject during the test and can be calculated by: DI = E + OHx 100.
TBTP-RT (work-efficiency)	Measure of both the attentional and perceptual abilities of the subject. It is related to the total score of hits (H), errors (E) and omissions (O) and it is calculated by: *WE=H−* (E + O). It is a measure of the subject’s work quality.
Trail-making test (A/B)	Attention and processing speed. TMT B also assess task switching.
TMT A (time)	Time used, in seconds, to perform the test. Trails A is just a psychomotor speed control for Trails B. In trails A you do not switch between numbers and letters, but just move from number to number connecting them through out the test.
TMT A (errors)	Errors made in carrying out the test.
TMT B (time)	Time used, in seconds, to perform the test. TMT-B measures task switching (the participant has to switch from numbers to letters as he/she does the task).
TMT B (errors)	Mistakes made in carrying out the test.
Wisconsin Card Sorting Test (WCST)	This test aims to assess abstract reasoning and the individual’s ability to generate problem-solving strategies in response to changing stimulation conditions. In general, your objective is to verify the individual’s performance in tasks that demand executive functions
WCST errors	Total number of errors.
WCST perseverance	When the subject persists in responding to a characteristic of the stimulus that is not correct, the response is considered a perseverance for that criterion and is scored as perseverative. The subject can persevere for the color, shape or number. Answers that meet the established perseverance criterion are considered perseverative, regardless of whether they are correct or incorrect. Responses that do not meet the perseverance criterion are “non-perseverative”.
WCST no perseverance errors	Corresponds to the answers that, being wrong, do not follow the preservation criterion by color, shape or number.
WCST perseverance errors	If the subject is making a category (eg, relating the bottom card to the top card) following the color criteria and you make a mistake (for example, you forget the category that was following—the color—and change to another—e.g. the shape), this error is counted as an error of set maintenance. If, on the other hand, the 10 trials with the same category are finished, the computer will change to another category. If the subject continues with the previous category, he will commit a perseverative error.
Rey-Osterrieth Complex Figure Test (FRey)	This test requires executive functions, in particular, organizational and planning skills [26]. The assessment of these skills implies an analysis of the subject’s performance, in terms of how he performs the reproduction of the figure and in terms of the strategies he uses.
FRey points (copy)	“FCR-Copy” index obtained through the application of the Osterrieth quotation system and proposed in the respective test manual (Rey, 1959/2002). This system consists of the evaluation of the 18 elements of the figure according to two criteria: the position of the element and the reproduction accuracy. The points awarded vary between 0 and 2 depending on the placement and accuracy of each element, with a maximum score of 36 points. 2 points are assigned for each correct and well positioned unit, 1 point for each correct but poorly positioned unit or which, although deformed or incomplete, is recognizable but is well positioned, and 0.5 points are attributed to the unit that is found misplaced and that is deformed or incomplete but recognizable. Finally, the unit that is unrecognizable or missing is quoted with 0 points.
FRey time (copy)	Time needed to make the copy.
FRey points (recall)	After a short break that should not exceed three minutes, the subject is invited to draw, on a second sheet, one second trial consisting of the reproduction of memory of the copied geometric figure. There is no time limit for playback; it’s the subject himself who will indicate when he has finished.
FRey time (recall)	Time needed to make second trial of the reproduction of Rey’s figure.
California Verbal Learning Test (CVLT)	This test evaluates episodic verbal learning and memory
CVLT A1	Free evocation of Monday’s list. Measures the capacity of information / stimulus coding.
CVLT A5	Free evocation of Monday’s list. Measures the performance of the information encoding after the 4 repetition sequences.
CVLT A1-A5	Free evocation of Monday’s list. Measures the ability to learn through repetition throughout the test.
CVLT B	Free evocation of Tuesday’s list. Objective interference after Monday’s list.

### Statistical analysis

All statistical analyses used the Statistical Package for the Social Sciences (SPSS) for Windows software, version 22.0, and the significance level was set at α = 0.05, i.e. statistical significance is achieved if *p* < 0.05. The demographic and clinical characteristics of the three groups were compared using the Chi-Square and ANOVA tests. The categorical variables were described as absolute values (n), and the normal distribution was assessed by the Kolmogorov–Smirnov test. An extensive comparison between the good-insight, poor-insight and control groups was conducted based on CVLT, TBTP, TMT, Rey’s figure test and WCST, using the ANOVA test to compare the mean scores between groups. Note that the level of education is a proxy measure for the intelligence level and that the statistical analysis of the education factor in the three groups proved statistically significant (*p* = 0.009). Since education level influences the results of the neuropsychological tests, an ANCOVA controlling for this factor was used (see Tables 3 and 4).

In order to control for the false discovery rate (FDR), we conducted a correction for multiple comparisons according to Benjamini and Hochberg [53]. By adjusting the computed *p*-values based on the FDR, we found that all previously computed *p*-values were statistically significant if lower than 0.029, i.e., the last rejected hypothesis was the variable “FRey points (late recall)” (see Table 3). Therefore, all other hypotheses with *p*-values lower than 0.05 but higher than 0.029 were not rejected, according to the aforementioned criterion.

The effect size was assessed with the Excel software, version 16.30, using the Cohen’s D statistic associated with the *t*-test for the comparison of means, the Cohen’s F statistic associated with ANOVA tests and the Cohen’s W statistic associated with the Chi-Squared independence tests.

## Results

The key results of this study can be divided into two parts: the first one comprises the results relating to the executive functions, summarized in Table 3, and the second part comprises those relating to memory functions (subserved by the hippocampus/temporal lobes), shown in Table 4. All results are based on the evaluation of 110 participants, divided into the control group (*n* = 53), the good-insight group (*n* = 37) and the poor-insight group (*n* = 20), please see Table 2.

**Table 2:** Demographic and clinical characteristics of the control, good-insight and poor-insight groups

	Control n/Mean ± SD	Good insight n/Mean ± SD	Poor insight n/Mean ± SD	Chi-Square/ F-test	***p***	Cohen’s D/F/W
**n**	53	37 (65%)	20 (35%)			
**Sex**						
**male**	23	19	15	5.814	0.055	W = 0.23
**female**	30	18	5			
**Age (years)**	33.0 ± 11.8	31.1 ± 11.7	33.1 ± 14.6	0.242	0.785	F = 0.07
**Education (years)**	14.2 ± 3.2	11.9 ± 2.6	13.2 ± 5.0	4.981	0.009	F = 0.31
**Mild depression**						
**yes**	1 (1.8%)	5 (13.5%)	4 (20%)	7.084	0.029	W = 0.25
**no**	52	32	16			
**Agoraphobia**						
**yes**	2 (3.7%)	2 (5.4%)	0 (0%)	1.088	0.580	
**no**	51	35	20			W = 0.10
**Schizoid personality**						
**yes**	0	0	1 (5.0%)	4.541	0.103	W = 0.20
**no**	53	37	19			
**Y-BOCS total score**	–	25.4 ± 13.3	23.7 ± 11.2	0.227	0.636	D = 0.13
**Y-BOCS obsession**	–	12.8 ± 6.7	12.4 ± 5.4	0.042	0.839	D = 0.06
**Y-BOCS compulsion**	–	12.7 ± 6.8	11.2 ± 6.5	0.576	0.451	D = 0.22
**Psychotropics**						
**yes**	0 (0.0%)	32 (86.5%)	16 (80.0%)	79.422	≤0.001	W = 0.85
**no**	53	5	2			

As shown in Table 2, the total number of patients (good-insight + poor-insight) is 57, which is higher than that of the control group [53]. With a false discovery rate of 0.029, the three groups did not differ significantly with regard to age, sex, Y-BOCS total score and compulsion and obsession scores. The *p* value for sex, 0.055, although close to the cut-off point (0.029), does not allow us to consider this variable as a relevant factor differentiating the three samples.

With *p* = 0.009, education (years) has proved a significant factor, and was therefore controlled afterwards with an ANCOVA (see Tables 3 and 4).

**Table 3:** Results of the executive function tests—WCST, TBTP and TMT—for the control, good-insight and poor-insight groups controlling for education (ANCOVA)

Executive/Attention						
**ANCOVA**						
Test	**Control** Mean ± St. Error	**OCD good insight** Mean ± St. Error	**OCD poor insight** Mean ± St. Error	**F-test**	***p***	**Cohen’s F**
TBTP-ID (dispersion index)	12.6 ± 1.9	19.0 ± 2.3	10.3 ± 3.1	3.243	0.043	0.25
TBTP-RT (work-efficiency)	199.3 ± 8.5	146.0 ± 10.3	171.2 ± 13.9	7.749	**0.001**	0.38
TMT A (time)	39.6 ± 3.3	50.6 ± 4.0	61.1 ± 5.5	6.101	**0.003**	0.34
TMT A (errors)	0.5 ± 0.1	0.1 ± 0.2	0.2 ± 0.2	2.203	0.116	0.20
TMT B (time)	87.7 ± 9.7	128.0 ± 11.6	148.5 ± 15.8	6.614	**0.002**	0.36
TMT B (errors)	1.7 ± 0.5	3.2 ± 0.6	2.5 ± 0.8	1.784	0.173	0.18
WCST errors	15.7 ± 1.4	17.6 ± 1.7	41.9 ± 2.3	52.502	**≤0.001**	1.00
WCST perseverance	9.2 ± 0.8	9.1 ± 1.0	31.8 ± 1.4	115.930	**≤0.001**	1.51
WCST no perseverance	8.1 ± 0.9	9.9 ± 1.1	10.3 ± 1.5	1.135	0.325	0.15
WCST perseverance errors	8.5 ± 0.7	7.7 ± 0.8	31.6 ± 1.1	178.916	**≤0.001**	1.87

**Table 4:** ANCOVA results of the memory tests—Rey’s figure test and CVLT—for the control, good-insight and poor-insight groups

Memory tests						
**ANCOVA**						
Test	**Control** Mean ± St. Error	**OCD good insight** Mean ± St. Error	**OCD poor insight** Mean ± St. Error	**F-test**	***p***	**Cohen’s F**
FRey points (copy)	31.0 ± 0.7	28.1 ± 0.9	30.8 ± 1.2	3.602	0.031	0.26
FRey time (copy)	2.5 ± 0.2	3.0 ± 0.2	3.6 ± 0.3	5.835	**0.004**	0.33
FRey points (recall)	20.1 ± 1.1	15.4 ± 1.3	20.2 ± 1.8	4.272	**0.016**	0.28
FRey time (recall)	2.3 ± 0.1	2.1 ± 0.2	2.8 ± 0.2	3.653	**0.029**	0.26
CVLT A1	5.7 ± 0.2	5.4 ± 0.3	5.1 ± 0.4	0.672	0.513	0.11
CVLT A5	11.7 ± 0.4	10.3 ± 0.4	11.2 ± 0.6	2.709	0.071	0.23
CVLT A1-A5	47.7 ± 1.4	42.0 ± 1.7	44.5 ± 2.3	3.292	0.041	0.25
CVLT B	5.6 ± 0.3	5.1 ± 0.3	6.1 ± 0.4	1.646	0.198	0.18

In what concerns the effects of pharmacological treatment on test performance, the consumption of the assessed pharmacological groups (antidepressants, benzodiazepines, antipsychotics, mood stabilizers, amphetamines) was identified only in the OCD group, with 32 psychotropic users in the good-insight subgroup and 16 in the poor-insight subgroup, corresponding to *p* ≤ 0.001. Therefore, the differences between the three groups are significant, as the use of psychotropic drugs was absent in the control group (0.0%) compared to both OCD groups (good and poor insight). The use of psychotropic drugs may affect the results of the neuropsychological tests. However, both OCD groups were found to use these drugs in similar percentages (86.5% vs 80%), which means that this variable cannot account for the neuropsychological results found.

Comorbidity was assessed using the MINI (based on DSM-IV-TR), as indicated in the Measurements section. Moreover, various disorders constituted exclusion criteria, including schizophrenia and bipolar affective disorder. Among those that were not excluded, three were present in the sample, namely mild depression, agoraphobia and schizoid personality disorder. Considering that the cut-off point established according to FDR was 0.029, two disorders did not exhibit significant differences in the two groups and were therefore excluded: agoraphobia (*p* = 0.580) and schizoid personality (*p* = 0.103). Mild depression (*p* = 0.029) was significantly more prevalent in the poor-insight group.

Table 3 shows the results of the tests on executive functions and attention. After the correction for multiple comparisons mentioned above, only the results with very low *p* remained: the “time” criterion of the TMT, which was more significant in TMT-B (time and errors), and the WCST’s different evaluation criteria. These results are relevant due to the magnitude of the differences found. See, for example, the “errors” variable in WCST: the errors of patients with poor insight are considerably higher than those of the other two groups. This fact, along with the increase in the TMT’s “time” criterion, is crucial for the debate on the neuropsychological profile of poor-insight patients, discussed in detail in the next section. For the ANCOVA tests presented in Table 3, the control group and the poor insight OCD group exhibited statistically significant differences in TMT A (time) and TMT B (time), in total WCST errors, WCST perseverance and in the WCST perseverance errors. The control group and the good insight OCD group showed statistically significant differences in the TBTP-RT (work-efficiency), TMT A (time) and TMT B (time). The good insight OCD group and the poor insight OCD group exhibited statistically significant differences in TBTP-RT (work-efficiency), total WCST errors, WCST perseverance and WCST perseverance errors.

Table 4 shows the variables used for assessing memory, i.e. the Rey’s figure and CVLT results controlled for education years. The differences concerning the Rey’s figure test (copy and 5-min recall) were the most significant. However, since the analysis was based on a multiple comparison, we resorted to the correction prescribed by Benjamini and Hochberg, as mentioned earlier. Following the correction, only the subscores with *p* ≤ 0.029 remained, i.e. FRey time (immediate recall), FRey time (late recall) and FRey points (late recall), which concern visual memory. None of the CVLT subscores were significant. For the ANCOVA tests presented in Table 4, the control group and the poor insight OCD exhibited statistically significant differences in FRey time (copy) and in FRey time (recall). The control group and the good insight OCD group showed statistically significant differences in the FRey time (copy) and FRey points (recall). The good insight and the poor insight OCD group exhibited statistically significant differences in FRey time (copy), FRey points (recall) and in FRey time (recall).

## Discussion

The main objective of this study was to determine whether there are relevant neuropsychological differences between good-insight and poor-insight OCD patients. It is already known that OCD patients have various neuropsychological deficits. They were found to have worse performances in many different studies [22], particularly concerning sustained attention, planning, response inhibition, set shifting, cognitive flexibility, verbal and non-verbal memory, visuospatial abilities, processing speed, working memory and special working memory.

We hypothesized that OCD patients with poor insight would have a worse performance on executive function tests (e.g. WCST and TMT) compared with OCD patients with good insight. The comparison between different neuropsychological studies is very difficult, since the test batteries are not the same and other study design characteristics also differ significantly, like the total number of patients or the methods used to collect the sample (studies using internet questionnaires and auto-filled tests tend to have larger samples).

As regards sustained attention, accuracy and fatigue resistance (TBTP work-efficiency (RT) score, see Table 3), poor-insight OCD patients showed a worse performance compared to the good-insight group. Given that poor insight corresponds to a more severe form of OCD, and that obsessions lead to a decrease in the patients’ reasoning speed, this finding demonstrates the cognitive difficulties faced by OCD patients, and particularly by those with poor insight. Previous studies have already considered the decrease in OCD patients’ task performance speed, rejecting the hypothesis that such a decrease is due to intrusive thoughts or meticulousness [54], and linking it instead to an inadequate precocious inhibition of competing ideas. However, it should be noted that these studies have not taken insight into account [54]. Still with regard to task switching and processing speed (TMT’s time criterion), we found clear differences between the three groups. Similar differences were also found in a meta-analysis including 115 studies [22], showing that OCD patients are indeed slower than controls.

Since TMT-B is more demanding than TMT-A, the former presented an even greater difference between the two groups (*p* = 0.002 vs *p* = 0.003*)*, highlighting the cognitive limitations of the poor-insight group, in particular regarding executive functions and not motor slowing. Given that poor-insight patients are more severely affected by OCD [55], it is plausible that their TMT performance is worse. Indeed, their difficulty in performing a task that requires continuous response inhibition and attentional set shifting is linked to the deficits of patients who do not respond to, or resist, treatment, namely poor-insight patients. The cognitive processes involved in TMT-B are varied, which implies that the cerebral regions involved are also varied [56]. In studies using voxel-wise lesion symptom mapping (VLSM), which investigates the neural correlates of a given lesion, it was concluded that the left rostral anterior cingulate was not only not exclusive to TMT, but also indicative of poorer WCST performance. This conclusion is particularly important in the present context, since the patients with poor insight we have analysed also fared worse in WCST (see Table 3). In sum, the regions involved in poor TMT-B performance were all located on the left side. Nevertheless, the conclusions to be drawn from TMT must take into account that the increase in the execution time is not linked to a specific pathology, as it was also recorded for schizophrenia, for example [57].

In the literature published in the last 10 years on the executive functions of OCD patients, which does not consider insight, researchers agree that the WCST is one of the tests in which OCD patients fare worst [58]. In our sample, the most common errors in the poor-insight group concerned perseverance (“perseverance errors”—WCST) and the patients’ difficulty in adapting their decisions to the results obtained during the test. Cognitive flexibility and set-shifting were clearly compromised among poor-insight patients (wcst total errors, wsct perseverance and perseverance errors) when compared to the two other groups (see Table 3), what is specifically due to impairments in cognitive flexibility but not to poor learning. Given the cognitive rigidity for which obsessive patients are known, this explanation is plausible and allows us to account for their insight from a neuroanatomical perspective, involving the pre-frontal and dorso-lateral cortices. From an anatomical perspective, these differences are in accordance with the neuroanatomical basis of obsessions and compulsions (orbito-frontal cortex, anterior cingulate cortex and caudate nucleus) proposed by many authors [4, 59–61]. Another study, with a smaller sample (14 patients), crossed the WCST results with the observation of the regional blood flow and concluded that the perseverance errors were related with flow changes in the right thalamus [62]. However, this study did not focus specifically on insight.

Although the neuropsychological characteristics of OCD have been regarded as a phenotypic marker [63], most results are inconsistent [58]. An important study comprising 150 OCD patients found significant changes in their WCST results when compared with those of the control group [27]. It was proposed that the increased checking behaviour, due to an effort to avoid making mistakes and an inability to spontaneously generate alternative solutions and organizational strategies, or simply indecision when evaluating and choosing between alternatives, may explain these findings [58]. Deficits in the executive functions (e.g. decision-making, response inhibition and cognitive flexibility) may be associated with insensitivity to the future consequences of patients’ choices and to defective planning in daily life [27, 64]. According to the authors who proposed this association, these differences can be explained by the patients’ organizational strategies. However, the reason for this has yet to be clarified, and not all studies point in the same direction [23, 53, 55, 56]. Finally, in a research completed in 2009, WCST scores were similar in different insight groups [33], but not in schizophrenic patients. This study proposed cognitive flexibility and set-shifting as “candidate” characteristics to what the authors call an “endophenotype of early-onset OCD” [65, 66] based on the WCST and TMT (among other) scores. In light of Tumkaya et al.’s conclusions and our own results, OCD patients with poor insight show some similarities in cognitive dysfunction to patients with schizophrenia [33].

As for visuospatial constructional abilities and visual memory (Rey’s complex figure test), these skills (associated with frontal lobe function [28, 59, 67]) are very important for daily life and for many different aspects of cognitive and visual capabilities [68]. The differences observed in the studied groups were statistically significant (see Table 4) and require a detailed analysis. If, on the one hand, patients with poor insight scored higher than those with good insight (which means that their copy was more accurate), the opposite is true when it comes to the time spent, measured by the F.Rey time copy and the F.Rey recall items. It is possible that patients with poor insight, although having provided better copies, spent more time drawing them. This result may be seen as a consequence of the dysfunction and the deficit in planning and organizational strategies exhibited by OCD patients [48, 63].

In what concerns verbal memory, the existing results have always been controversial, and our study is no exception [22]. While no differences were found on the CVLT, some links between verbal memory and poor insight in OCD were recorded [28]. To the best of our knowledge, no real consensus has been reached as to what these findings mean [32]. It was also proposed that verbal memory is not affected by OCD [27, 58, 63, 69]. These studies argue that verbal memory is not compromised, but rather the patients’ “organizational strategies” [63]. Deckersbach et al. proposed that OCD patients underutilize organizational strategies rather than lacking verbal memory per se [70]. The fact that verbal memory was not significantly different in the good-insight and poor-insight groups suggests that what differentiates OCD patients is not memory, which is preserved in this disease [55], but more complex functions, such as the executive functions discussed above.

In order to secure the homogeneity of our sample and avoid biases, several comorbities were excluded from our study, such as schizophrenia, bipolar affective disorder and mental retardation. Of those that were included, according to the MINI’s criteria, three were found in the sample: schizotypal personality disorder, agoraphobia and mild depression (see Table 2). Only one case of schizotypy was found, in the poor-insight group. This fact, although lacking statistical relevance, is plausible, given the link between schizotypy and insight [71]. Indeed, several bibliographic sources point to a correlation between OCD with poor insight, and hence with worse prognosis, and schizotypal disorders [5]. However, we have not used a specific scale to measure schizotypy, which might have yielded different results. Agoraphobia, a fairly frequent disorder, was also statistically insignificant, with only four cases in the whole sample—two in the good-insight group and two in the control group. Although many authors claim that patients with poor insight display more comorbidities, recent studies have also failed to identify statistically relevant values of agoraphobia in OCD groups with good and poor insight [72], which suggests, for now, that the comorbidity of OCD with agoraphobia is not a research priority. As for depression, the percentage of patients suffering from this condition was higher in the poor-insight group (with *p* = 0.029, which coincides with the cut-off value, and a low effect size (0.25)). In our view, since the absolute quantity of individuals with depression was less than 5, any conclusions based on a statistical analysis of these data would be biased. Therefore, we leave the investigation of this aspect to future studies with larger subsamples.

Finally, it is worth mentioning the main limitations of this research. However, insight assessment changes over time, limiting the research of this issue. Our study is cross-sectional, which means we have not taken into account changes in symptomatology and insight in the course of time, unlike other authors [73]. This limits the depth of our analysis. Moreover, although the scale we have used to measure insight, BABS, enables good comparisons with other studies on insight, it is based only on patients’ pre-existing and explicitly held beliefs [35, 74], which limits its results. As regards the sample size, although it is similar to that of other studies, future research should aim for larger samples so as to reach broader conclusions with stronger *p* values.

## Conclusions

The overall direction of our results leads us to conclude that OCD patients with poor insight have a significantly worse neuropsychological performance than the good-insight and control groups. In summary, poor-insight patients have greater neuropsychological dysfunction than those with good insight, especially regarding the executive functions. On the contrary, verbal memory does not seem to differ in the three groups. Moreover, we believe that a neuropsychological evaluation of poor-insight patients is crucial for their future and clinical outcome, particularly if it leads to the development of targeted neurocognitive therapies.

Future work in this field will require larger sample sizes, the inclusion of functional imaging examination and a longitudinal clinical evaluation.

## Supplementary Information

**Additional file 1.**

## Data Availability

The data supporting the results reported in the article are available from the corresponding author upon reasonable request.

## Abbreviations

BABS: Brown Assessment of Beliefs Scale
CVLT: California Verbal Learning Test
F-Rey: Rey-Osterrieth Complex Figure Test
MINI: Mini International Neuropsychiatric Interview
OCD: Obsessive-compulsive disorder
TBTP: Toulouse–Piéron test
TMT: Trail-making test
WCST: Wisconsin Card Sorting Test
Y-BOCS: Yale-Brown Obsessive-compulsive scale
