# Supplementary material for: Executive functions and insight in OCD: a comparative study
Source: BMC Psychiatry. 2021 Apr 29;21:216. doi: 10.1186/s12888-021-03227-w (PMC8082868; doi:10.1186/s12888-021-03227-w)
Supplement: Supplementary file 1 — Additional file 1. [file 12888_2021_3227_MOESM1_ESM.doc]

Title

Executive functions and insight in OCD: a comparative study.

Authors’ names and affiliations

Lucas Manarte.

Av. Prof. Egas Moniz, 1649-028 Lisboa, Portugal ^a^

António R. Andrade.

Av. Rovisco Pais 1, 1049-001 Lisboa, Portugal ^b^

Linete do Rosário.

Av. Prof. Egas Moniz, 1649-028 Lisboa, Portugal ^a^

Daniel Sampaio.

Av. Prof. Egas Moniz, 1649-028 Lisboa, Portugal ^a^

Maria Luísa Figueira.

Av. Prof. Egas Moniz, 1649-028 Lisboa, Portugal ^a^

Pedro Morgado.

R. da Universidade, 4710-057. Braga, Portugal ^c^

Barbara J. Sahakian. Herchel Smith Building for Brain & Mind Sciences, Forvie Site, Robinson Way. Cambridge CB2 0SZ, UK ^d^.

^a^ Psychiatry and Mental Health Department, Faculty of Medicine of the University of Lisbon

^b^ IDMEC. Instituto Superior Técnico. University of Lisbon

^c^ Life and Health Sciences Research Institute, School of Medicine, University of Minho

^d^ Department of Psychiatry and Behavioural and Clinical Neuroscience Institute, University of Cambridge

**Corresponding author**

Lucas Manarte. E-mail: [manarte@campus.ul.pt](mailto:manarte@campus.ul.pt). ORCID: <https://orcid.org/0000-0002-9771-8179>

**Appendix**

**Title:**

**Validation of the Portuguese version of the Brown Assessment of Beliefs Scale: Psychometric properties and comparison with other studies**

**Introduction**

The Brown Assessment of Beliefs Scale (BABS) has been used in psychological assessments, namely in the assessment of obsessive-compulsive and body dysmorphic disorders. BABS is a clinician-administered seven-item scale designed to assess delusions across a wide range of psychiatric disorders^1^. The score for each item ranges from 0 (non-delusional, or least pathological) to 4 (delusional, or most pathological). The scale’s original version had fifteen items, that were later reduced to seven: i) conviction; ii) perception of other people’s views of one’s beliefs; iii) explanation of different views; iv) fixity of ideas; v) attempt to disprove beliefs; vi) insight; and vii) ideas/delusions of reference. This last item is not included in the scale’s total score, as it is not considered relevant to all disorders characterized by delusional thinking.

It is currently debated whether delusions are a dichotomous quantity or a continuum construct of insight spanning from good to poor^1^. Note, however, that as the BABS total score (without considering the seventh item) ranges from 0 (no delusional thinking) to 24 (complete lack of insight), it provides a continuum range of insight rather than a dichotomous measure.

BABS was initially used for three diagnoses: obsessive-compulsive disorder (OCD), body dysmorphic disorder (BDD) and psychotic mood disorder (PMS). Although BABS had good psychometric properties (e.g. interrater reliability, test-retest reliability, internal consistency), the samples were quite small: n=20 for OCD, n=20 for BDD and n=10 for PMS. Later, further analysis was conducted with larger samples, namely by Eisen et al. (2001)^2^, with a sample size of n=71 for OCD, by Eisen et al. (2004)^3^, with a sample size of n=64 for OCD and n=85 for BDD, and more recently by Phillips et al. (2013)^4^, with a sample size of n=327 for BDD. In all these studies, BABS’ one-factor structure was validated with very good psychometric properties.

According to Eisen et al. (1998), the BABS metric has been widely used in OCD and BDD studies, mainly due to its brevity, ease of administration and the fact that it provides a continuous measure of delusion (instead of a dichotomous measure). However, different classification rules for good insight vs. poor insight have been proposed and contrasted with the gold standard rating from the Yale–Brown Obsessive Compulsive Scale (Y-BOCS), assessing the sensitivity and specificity of different cut-off points.

More recently, translations and validations of BABS in German^5^ and in Chinese^6^ were published. In the German validation, the original BABS was translated, back-translated and administered to 70 individuals diagnosed with BDD; in the Chinese validation, it was translated separately by two bilingual translators and administered to 171 individuals diagnosed with OCD. Both validations exhibited good psychometric properties and supported the original one-factor structure. An Exploratory Factor Analysis (EFA) was used in the German validation and a Confirmatory Factor Analysis (CFA) in the Chinese validation.

Moreover, the convergent validity of the BABS measure has been assessed with other measures, namely the Yale-Brown Obsessive-Compulsive Scale (Y-BOCS) and its extension for BDD patients (BDD-YBOCS), leading to the discovery of statistically significant correlations with the insight item of the Y-BOCS scale^5,6^.

**Objective**

The present study aims to develop, translate and validate BABS for Portuguese, as well as to assess its psychometric properties, namely its internal consistency, interrater reliability and test-retest reliability.

**Methods**

This study was approved by the Ethics Committee of the Northern Lisbon Hospital Centre – Santa Maria Hospital and by the Scientific Committee of the Faculty of Medicine of the University of Lisbon.

The English version of the BABS questionnaire was translated into Portuguese by a team of professional translators. The Portuguese translation was conducted in accordance with the standardized back-translation procedure in order to guarantee the semantic equivalence to the original BABS^1^ . The procedure involved two translations: the first one from the original English version into Portuguese and the second one from Portuguese back into English. The latter was then compared with the original English version in order to discuss the existing discrepancies and to reach a final consensus, which was then taken into account for the final Portuguese version. Each step of the process was conducted by a separate translator.

A sample of psychiatric patients was used to conduct the Exploratory Factor Analysis (EFA) and Confirmatory Factor Analysis (CFA). The original one-factor model was validated, reporting the reliability statistics (e.g. internal consistency, interrater reliability and test-retest reliability) and the EFA and CFA associated statistics.

The sample was composed of patients from private practices and from Santa Maria Hospital, in Lisbon.

Table 1 – Descriptive statistics and correlation of individual scores with total scores without the item score.

| Scale item | Item score | | Correlation of item score with total score minus the item score (n=157) | | Interrater reliability ICC (n=10) |
| --- | --- | --- | --- | --- | --- |
|  | Mean | SD | r | P | ICC |
| Conviction | 2.09 | 1.25 | 0.64 | <0.001 | 0.94 |
| Perception of others people’s views of one’s beliefs | 2.07 | 1.06 | 0.47 | <0.001 | 0.93 |
| Explanation of different views | 2.06 | 1.16 | 0.69 | <0.001 | 1.00 |
| Fixity of ideas | 1.60 | 1.27 | 0.48 | <0.001 | 0.96 |
| Attempt to disprove beliefs | 2.38 | 1.49 | 0.30 | <0.001 | 0.99 |
| Insights | 2.17 | 1.35 | 0.30 | <0.001 | 1.00 |
| Ideas/delusions of reference | 1.09 | 1.00 |  |  | 0.98 |
| BABS (total) | 12.37 | 4.96 |  |  | 1.00 |

The Exploratory Factor Analysis with a Kaiser–Meyer Olkin measure of 0.771 indicated a reasonable adequacy of the one-factor model, which explains the total of 46% of the variability. This value is low when compared to other studies: 56% in Eisen et al. (1998)^1^, 60% in Phillips et al. (2013)^4^ and 72% in Buhlmann (2014)^5^.

**Confirmatory Factor Analysis in AMOS**

Figure 1 – Single-factor structure of the BABS with the standardized loadings of each item from P1 to P6.


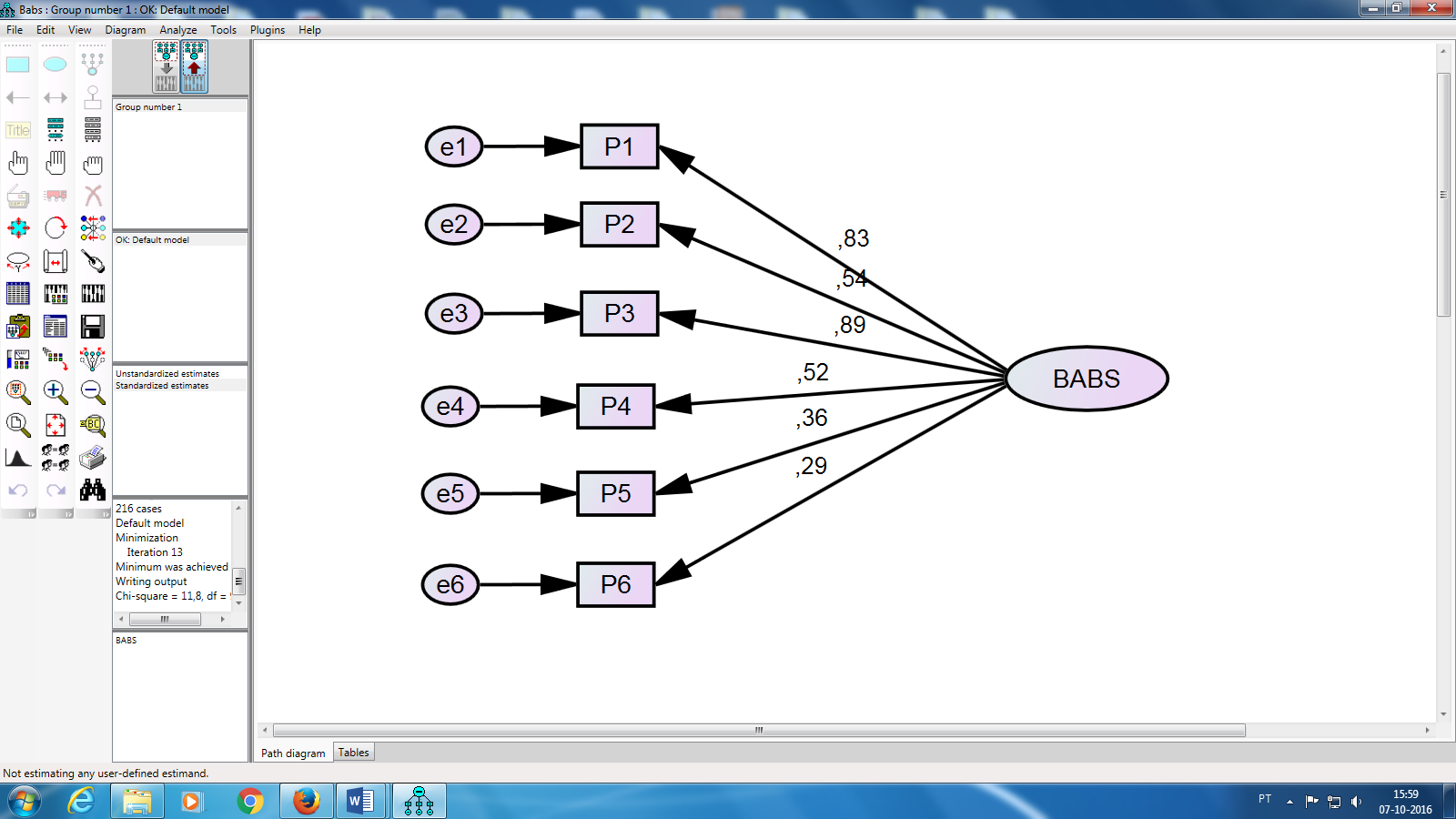


The loadings are all statistically significant (p<0.001) and fell within the range 0.29–0.89, but are lower than the ones found in Niu et al. (2016)^6^, from 0.36 up to 0.95.

The Confirmatory Factor Analysis showed that the single latent factor structure has a reasonably good fit, with adequate fit indices: χ2/df=1.319, CFI=0.991 and RMSEA=0.038. These three fit indices are: i) the Chi-Square statistic divided by the number of degrees of freedom (χ2/df); ii) the Comparative Fit Index (CFI); and iii) the Root Mean Square Error of Approximation (RMSEA). In practice, for the Chi-Square statistic divided by the number of degrees of freedom, ratios lower than 3 still represent a good data model fit, whereas for the CFI, the closer they are to 1 the better the data fits the model. Finally, the RMSEA should be as close to 0 as possible. In this case, the value 0.10 is usually regarded as a cut-off point for poor fitting models. Test-retest reliability was assessed by administering BABS twice to the same patients.

**Internal consistency**

The internal reliability of BABS was not excellent (<0.90), but it is in line with the literature, with a Cronbach’s alpha of 0.729. If an item is excluded, Cronbach’s alphas range from 0.629 to 0.750.

The exploratory and confirmatory factor analyses supports the original one-factor structure, with a KMO=0.771, a total of 46% of variability captured in this single factor for the EFA and reasonably good fit indices: χ2/df=1.319, CFI=0.991, and RMSEA=0.038 for the CFA.

**Conclusion**

The sample showed that BABS is a valid instrument to measure the patients’ insight concerning their delusions, with good psychometric properties.

**References**

1. JL E, KA P, Baer L, DA B, KD A, SA R. The Brown Assessment of Beliefs Scale: Reliability and validity. *Am J Psychiatry*. 1998;155(1):102-108. doi:10.1176/ajp.155.1.102

2. Eisen JL, Rasmussen SA, Phillips KA, et al. Insight and treatment outcome in obsessive-compulsive disorder. *Compr Psychiatry*. 2001;42(6):494-497. doi:10.1053/comp.2001.27898

3. Eisen JL, Phillips KA, Coles ME, Rasmussen SA. Insight in Obsessive Compulsive Disorder and Body Dysmorphic Disorder. *Compr Psychiatry*. 2004;45(1):10-15. doi:10.1016/j.comppsych.2003.09.010

4. Phillips KA, Hart AS, Menard W, Eisen JL. Psychometric evaluation of the brown assessment of beliefs scale in body dysmorphic disorder. *J Nerv Ment Dis*. 2013;201(7):640-643. doi:10.1097/NMD.0b013e3182983041

5. Buhlmann U. The German version of the Brown Assessment of Beliefs Scale (BABS): Development and evaluation of its psychometric properties. *Compr Psychiatry*. 2014;55(8):1968-1971. doi:10.1016/j.comppsych.2014.07.011

6. Niu C, Liu W, Lei H, et al. The Chinese version of the Brown Assessment of Beliefs Scale: Psychometric properties and utility in obsessive compulsive disorder. *J Obsessive Compuls Relat Disord*. 2016;11:39-42. doi:10.1016/j.jocrd.2016.08.003
